# The Suitability of the South African Triage Scale (SATS) in Triaging Patients With Penetrating Neck Injuries at a High‐Level Trauma Center, in the Western Cape, South Africa

**DOI:** 10.1002/wjs.12578

**Published:** 2025-04-08

**Authors:** Thomas Steve Jessop, Lindi Martin, Hendrick J. Lategan, Julie M. Dixon, Nee‐Kofi Moul‐Millman, Elmin Steyn

**Affiliations:** ^1^ Division of Surgery Department of Surgical Sciences Stellenbosch University Faculty of Medicine and Health Sciences Cape Town South Africa; ^2^ Department of Emergency Medicine University of Colorado Denver Denver Colorado USA

## Abstract

**Background:**

Penetrating injuries are the most common mechanism of serious injury in Cape Town, with penetrating neck injuries (PNIs) having a mortality rate of 10%. The South African Triage Scale (SATS) is commonly used and designed for general emergency departments in South Africa. This study aimed to assess the suitability of the SATS for triaging patients with PNIs at a high‐level trauma center, Tygerberg Hospital (TBH).

**Methods:**

This secondary analysis utilized data from a multicentre prehospital observational study. Adult patients (≥ 18 years) with PNIs managed at the TBH Trauma Center between October 2022 and March 2023 were included. Patients dead on arrival were excluded. The original arrival triage categorization was reviewed and re‐calculated based on a correct application of the SATS.

**Results:**

Seventy patients (mean age: 32 years, SD: 10) were included. Mechanisms of injury were stab or cut (78.6%) and gunshot wound (21.4%). The most common SATS colors recorded were Orange (61.4%), and Yellow (17.1%), with recalculation of SATS (R‐SATS) resulting in “Orange” (81.4%) and “Red” (18.6%). Under‐triage occurred in 25.7% of cases. A significant difference was noted between SATS and R‐SATS categories (*p* < 0.01). There was no significant association between TBH SATS or R‐SATS category and need for resuscitation, urgent surgical intervention, and 7‐day mortality (*p* > 0.05).

**Conclusion:**

No association could be shown between the SATS colors and life‐saving interventions and mortality. Furthermore, findings suggest that the SATS is not optimally applied at TBH. Consideration for a simpler, better‐performing tool to optimize triage of patients with traumatic injuries, including PNIs is needed.

## Graphical abstract

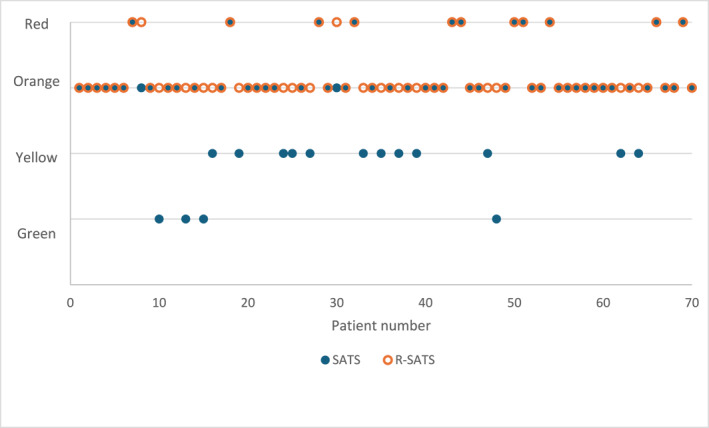

## 1 Background

Low‐ and middle‐income countries (LMICs) contribute a disproportionately large share of the global burden of disease of trauma [1]. Over 90% of global trauma‐related deaths occur in LMICs with healthcare systems characterized by limited resources and an overwhelming number of injuries [1, 2]. In Cape Town, South Africa (SA), injury due to interpersonal violence is rife, primarily affecting young men [2]. In SA trauma patients, those presenting to hospital with intentional injury are double the number presenting with unintentional injury [3].

Penetrating trauma (i.e., firearm or stab wounds) is the most common mechanism of serious injury (62.8%) presenting to regional referral trauma centers in Cape Town [2]. Due to the complex anatomical structure of the neck, a poor correlation exists between the external wound location and internal injuries [4]. Notably, the mortality rate associated with Penetrating Neck Injuries (PNIs) is high, at 10% [4].

Triage is a critical process in all emergency centers aimed at prioritizing patients based on the seriousness of their clinical condition to improve timeliness of care, resource utilization, and patient outcomes [5]. Lack of a triage system results in longer waiting times, poor management of high‐risk cases and increased morbidity and mortality [6]. Both under‐ and over‐triage are associated with increased morbidity and mortality [7]. The rate of mis‐triage in SA has been reported as 31.7% [8]. Of those mis‐triaged, 56% are under‐triaged, while 44% are over‐triaged. Similarly, under‐triage in trauma patients in high‐income countries (HICs), is estimated to range between 1% and 71%, underscoring the call for enhancements of trauma triage processes [7].

Worldwide, commonly evaluated triage systems include the Canadian Triage and Acuity Scale (CTAS), the Emergency Severity Index (ESI), the Manchester Triage System (MTS) and the South African Triage Scale (SATS) [9, 10]. The SATS is mostly used in LMICs (e.g., South Africa, Haiti, Afghanistan, and Pakistan) but has also been modified for use in HICs (Norway) [5, 11, 12, 13]. Notably, these systems show only moderate to good validity in categorizing patients as high or low levels of urgency [10]. The CTAS, ESI, and MTS were developed in and for high‐resource settings, whereas the SATS was designed for use in LMIC settings as a nurse‐led system [8, 9, 14].

The SATS is commonly used throughout healthcare facilities in SA and is used as a paper‐based system at Tygerberg Hospital (TBH), a tertiary care trauma center in Cape Town [8, 14]. Despite the availability of many internationally developed triage systems, their high complexity and increased time to compute render them unsuitable for the SA context characterized by limited resources, higher trauma patient numbers and delayed presentation leading to more advanced pathology [6]. The SATS has been found to have good validity in LMICs emergency settings for all‐comer undifferentiated conditions, however, it has not been validated to determine acuity in specialized trauma‐only settings compared to general emergency centers [5, 14].

SATS triages patients into color‐coded priority groups according to their medical acuity [15]. SATS categorizes patients as Green (non‐urgent), Yellow (urgent), Orange (very urgent) and Red (emergency) [14]. It consists of a two‐tiered approach; a series of discriminators and a physiological scoring system [the Triage Early Warning Score (TEWS)] [6]. The discriminators (a list of medical and traumatic conditions determined by local expert consensus) are categorized into Red (emergency signs), Orange (very urgent signs) and Yellow (urgent signs) [6]. Amongst the signs designated as Orange are “Mechanism of injury (high energy transfer)” and “Stabbed neck.” The discriminator signs should be used first to categorize a patient, however, if no signs are evident, the vitals are measured to calculate the TEWS [16]. The TEWS is the physiological scoring system and uses systolic blood pressure, heart rate, temperature, respiratory rate and Alert/Verbal/Pain/Unresponsiveness (AVPU) (to assess level of consciousness), mobility and a trauma factor (trauma present or not) to provide a score on a scale of 0 (lowest acuity) to 17 (highest acuity) [16].

Once a color has been allocated, the intention is that Red cases need immediate attention, Orange can wait 30 min or less, Yellow need to be assessed by the medical team within an hour and Green can wait for 2 h [16]. Admission criteria for the resuscitation area of the TBH Trauma Center is either a Red SATS score or a senior practitioner's clinical decision [17]. Notably, a study conducted at Khayelitsha Hospital, a hospital in a region with a high burden of penetrating injuries, found that almost 30% of patients requiring resuscitation had been under‐triaged as Green or Yellow [17].

The TBH Trauma Center is a US Level One equivalent center that mostly receives trauma patients transported from other facilities and very few directly from the scene of injury. Of referred patients, most have received some form of initial resuscitation. However, SATS is a triage tool designed to be used at the point of initial health system contact. Although patients are initially triaged at the referring facility, they need to be triaged again due to transfer delays (in the LMIC healthcare setting) and physiological deterioration (ongoing bleeding or evolving shock). Triage at TBH is not primary triage, but SATS is utilized to assist with prioritization of the large numbers of injured patients waiting to receive specialist care—a task SATS was not designed for.

There is currently no triaging system specifically for trauma emergencies available in SA and the value of the SATS in trauma‐only settings has not been adequately assessed [5]. In addition, the SATS has not been validated for penetrating injuries (e.g., PNIs), specifically not in a high‐level trauma center setting. This study aimed to determine the suitability of the SATS for triaging patients with PNIs at the TBH Trauma Center. To determine the suitability, both the accuracy of application by the triage staff as well as the validity of the triage score in predicting urgency for medical care, in patients with PNIs, need to be determined. The objectives were to determine (1) the accuracy of application of the triage scoring system to patients with PNIs and (2) the association between the SATS category and need for resuscitation, urgent surgical intervention and mortality.

## 2 Methods

This was a secondary analysis of data collected by a prospective, observational, multi‐center, cross‐sectional study entitled, “Epidemiology and Outcomes of Prolonged Trauma Care (EpiC): A Multicentre Prehospital Observational Study in the Western Cape” (ethics reference number: N20/03/036). We included all adult patients with PNIs managed at the TBH Trauma Unit between October 2022 and March 2023. Inclusion criteria comprised all patients triaged SATS Yellow or higher at the initial receiving facility. Exclusion criteria comprised patients younger than 18 years and those dead on arrival.

Data collected included patient demographics (age, sex), injury variables (force type, mechanism, anatomic data), all variables included in the TBH SATS, and criteria reflecting the severity of injury (i.e. need for resuscitation and urgent interventions). EpiC data collectors correct TEWS points calculation errors, however, allocation of TEWS points by triage staff was recorded. The TEWS and SATS categories were retrospectively recalculated to provide a Recalculated Triage Classification according to the SATS (R‐SATS) and TEWS (R‐TEWS) and compared with the original TEWS and SATS categories.

Statistical analyses were performed using SPSS V29 [18]. Descriptive statistics (e.g., means and standard deviations or medians and interquartile ranges (IQR), where appropriate, and counts and percentages) were computed to describe continuous and categorical data. The related‐samples Wilcoxon Signed Rank Test and the McNemar test were used to determine whether the patients with PNIs were accurately triaged. The association between the SATS category and need for resuscitation, urgent surgical intervention and early mortality was assessed using Pearson's Chi‐square test and Fisher's Exact Test, where appropriate. Statistical significance was set at *p* < 0.05.

## 3 Results

Seventy patients (mean age: 32.6 years, SD: 10.3; range: 18–60) were included, of which 92.9% (*n* = 65) were male. Table 1 represents demographic and injury information of the included patients. Over half (51.4%) were referred from a Level Three US equivalent, 40% from a Level Two US equivalent and 8.6% presented directly from the scene. Mechanisms of injury to the neck included stab or cut (78.6%) and gunshot wounds (21.4%).

**TABLE 1:** Demographics, mechanism of injury, SATS discriminators and disposal status.

Category		*N* (%)
Sex	Male	65 (92.9)
	Female	5 (7.1)
Mechanism of injury	Stab or cut	55 (78.6)
	Gunshot wound	15 (21.4)
SATS discriminator	Stabbed neck	55 (78.6)
	High energy transfer (severe mechanism of injury)	15 (21.4)
Arrived at TBH from	Level three equivalent	36 (51.4)
	Level two equivalent	28 (40)
	Presented directly	6 (8.6)
Trauma center initial resuscitation	Yes	6
	No	64
Number of operating theater encounters	0	64
	1	5
	2	1
7‐Day mortality	Yes	1
	No	69
Disposal status from hospital	Discharged home (independent)	7 (10)
	Discharged home (followed up at lower level of care facility)	40 (57.1)
	Discharged home (with follow up at specialty care)	15 (21.4)
	Transferred (other facility)	7 (10)
	Deceased	1 (1.4)

The most common SATS color recorded was Orange (61.4%), followed by Yellow (17.1%) (Table 2). The most common TEWS color recorded was Yellow (41.4%) followed by Green (34.3%). The most common R‐SATS color was Orange (81.4%) followed by Red (18.6%). The most common R‐TEWS color was Yellow (47.1%), followed by Orange (40%).

**TABLE 2:** TEWS and SATS colors recorded by the TBH trauma unit and recalculated TEWS and SATS colors.

Color	SATS (*n*, %)	TEWS (*n*, %)	R‐SATS (*n*, %)	R‐TEWS (*n*, %)
Green	4 (5.7)	24 (34.3)	0	0
Yellow	12 (17.1)	29 (41.4)	0	33 (47.1)
Orange	43 (61.4)	9 (12.9)	57 (81.4)	28 (40)
Red	11 (15.7)	8 (11.4)	13 (18.6)	9 (12.9)
Total	70 (100)	70 (100)	70 (100)	70 (100)

**FIGURE 1:**
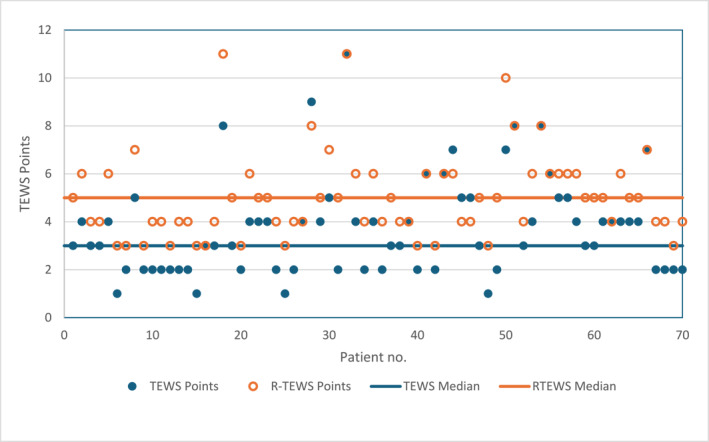
TEWS versus R‐TEWS points.

Figure 1 represents a scatterplot of original TEWS points versus R‐TEWS points. There was a significant difference in the TBH TEWS points and the R‐TEWS points (*p* < 0.001), with the latter being significantly higher than the original scoring by the triage staff (TEWS median: 3, IQR: 2–4 vs. R‐TEWS median: 5, IQR: 4–6).

The documented TEWS color and the R‐TEWS Color are presented in Table 3. There was a significant difference between the TEWS and R‐TEWS color assignment (*p* < 0.001). Over a quarter (28.6%) of patients were under‐triaged Yellow instead of Orange by the triage staff. A tenth (10%) of patients were correctly calculated TEWS Red. No patients were R‐TEWS Green.

**TABLE 3:** TEWS color versus recalculated‐TEWS color.

	Recalculated TEWS color					
TEWS color		Green (%)	Yellow (%)	Orange (%)	Red (%)	Total (%)
	Green	0	22 (31.4)	2 (2.9)	0	24 (34.3)
	Yellow	0	9 (12.9)	20 (28.6)	0	29 (41.4)
	Orange	0	2 (2.9)	5 (7.1)	2 (2.9)	9 (12.9)
	Red	0	0	1 (1.4)	7 (10.0)	8 (11.4)
	Total	0	33 (47.1)	28 (40)	9 (12.9)	70 (100)

*Note:* All those below the gray diagonal line represent patients over‐triaged, while those above the gray line represent patients under‐triaged.

Table 4 represents a cross‐tabulation of the original SATS colors versus R‐SATS colors and shows which patients were incorrectly triaged by the TBH triage staff. A statistically significant difference was determined between the SATS and R‐SATS color assignment (*p* < 0.001).

**TABLE 4:** SATS color versus R‐SATS color.

	R‐SATS color					
SATS color		Green (%)	Yellow (%)	Orange (%)	Red (%)	Total (%)
	Green	0	0	4 (5.7)	0	4 (5.7)
	Yellow	0	0	12 (17.1)	0	11 (15.7)
	Orange	0	0	41 (58.6)	2 (2.9)	43 (61.4)
	Red	0	0	0	11 (15.7)	11 (15.7)
	Total	0	0	57 (81.4)	13 (18.6)	70 (100)

*Note:* All those above the gray line represent patients under‐triaged. There were no patients below the gray line (over‐triage).

Table 5 represents the correct versus incorrect TEWS and SATS Color assignment. According to TEWS, most patients (65.7%, *n* = 46) were under‐triaged, while 4.3% (*n* = 3) were over‐triaged and 30% (*n* = 21) were correctly triaged. When using SATS, 25.7% (*n* = 18) of patients with PNIs were under‐triaged. A fifth (20%) by one category and 5.7% by two categories. No patients were over‐triaged.

**TABLE 5:** TEWS color for patients with PNIs.

			TEWS *N* (%)	SATS *N* (%)
Correct			21 (30)	52 (74.3)
Incorrect	Total		49 (70)	18 (25.7)
	Over‐triaged		3 (4.3)	0 (0)
		By 1 category	3 (4.3)	0 (0)
	Under‐triaged		46 (65.7)	18 (25.7)
		By 1 category	44 (62.8)	14 (20)
		By 2 categories	2 (2.9)	4 (5.7)

**FIGURE 2:**
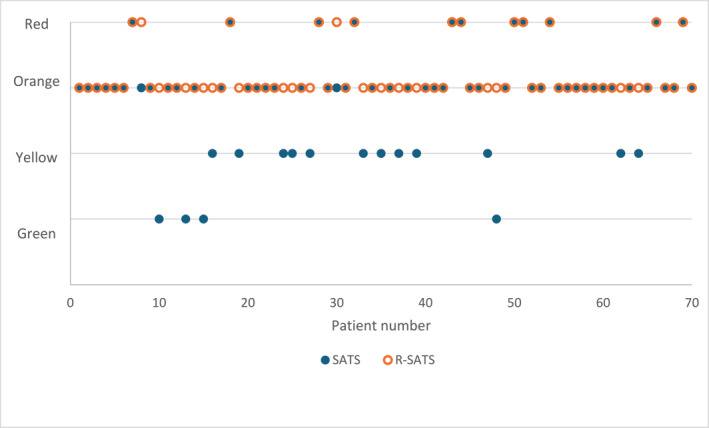
SATS versus R‐SATS color per patient.

Figure 2 is a graphical representation of the SATS colors versus R‐SATS colors for each patient, showing which patients were incorrectly triaged by the TBH triage staff.

Table 6 represents a cross‐tabulation between SATS and R‐SATS colors and those patients that needed resuscitation, the number of operating theater encounters and death within 7‐day. There was no significant association between the TBH initial SATS category and (1) the need for resuscitation, (2) urgent surgical intervention and (3) 7‐day mortality (*p* > 0.05). There was no significant association between the R‐SATS category and (1) the need for resuscitation and (2) urgent surgical intervention and (3) 7‐day mortality (*p* > 0.05).

**TABLE 6:** SATS and R‐SATS color versus needed resuscitation, number of operating theater encounters and death within 7‐days.

		Green	Yellow	Orange	Red	Total
SATS						
Needed resuscitation	No	4 (5.7)	12 (17.1)	40 (57.1)	8 (11.4)	
	Yes	0	0	3 (4.3)	3 (4.3)	6 (8.6)
	Fisher‐Freeman‐Halton exact test				0.104	
Number of operating theater encounters	0	4 (5.7)	12 (17.1)	40 (57.1)	8 (11.4)	64 (91.4)
	1	0	0	2 (2.9)	3 (4.3)	5 (7.1)
	2	0	0	1 (1.4)	0	1 (1.4)
	Fisher‐Freeman‐Halton exact test				0.199	
Death within 7‐day	Yes	0	0	0	1 (1.4)	1 (1.4)
	No	4 (6)	12 (17)	43 (61.4)	10 (14.3)	69 (98.6)
	Fisher's exact test				0.186	
RSATS						
Needed resuscitation		Green	Yellow	Orange	Red	Total
	No	0 (0)	0 (0)	54 (77.1)	10 (14.3)	64 (91.4)
	Yes	0 (0)	0 (0)	3 (4.3)	3 (4.3)	6 (8.6)
	Fisher's exact test				0.073	
Number of operating theater encounters	0	0 (0)	0 (0)	54 (77.1)	10 (14.3)	64 (91.4)
	1	0 (0)	0 (0)	2 (2.9)	3 (4.3)	5 (7.1)
	2	0 (0)	0 (0)	1 (1.4)	0	1 (1.4)
	Fisher‐Freeman‐Halton exact test				0.073	
Death within 7‐day	Yes	0 (0)	0 (0)	0 (0)	1 (1.4)	1 (1.4)
	No	0 (0)	0 (0)	57 (81.4)	12 (17.1)	69
	Fisher's exact test				0.186	

## 4 Discussion

Among a cohort of predominantly referred patients with PNI seen at the TBH tertiary trauma care referral center, this study found poor accuracy of the application of the SATS triage scoring system. Over a quarter (25.7%) of patients with PNIs were under‐triaged, a fifth (20%) by 1 SATS category and 5.7% by 2 SATS categories.

In a healthcare setting with an overwhelming trauma burden, under‐triage has the potential to negatively impact patient care and outcomes by causing delays to care and inadequate resources to be allocated to such patients. At the TBH Trauma Center, under‐triage can be explained by poor staff training and poor replication of SATS onto the triage paper stationery, both clarified below.

A high‐level general emergency center in Johannesburg, SA, reported that failure to record a discriminator, occurring in 45.7% of mis‐triage cases, was the main reason for triaging errors [8]. While the TBH Trauma Center triage documents allow for the discriminators to be documented, the discriminator list is not printed. Although there is ongoing retraining in SATS, documentation of the discriminators relies on the triage staff being well‐trained and informed on which discriminators apply to the individual patients (e.g., triage nurses may not associate a gunshot wound to the neck as a “Mechanism of Injury—High energy transfer”), and the color upgrade associated with the discriminator used. Findings suggest documentation and color assignment of discriminators are not correctly done as we would expect no cases to have a SATS of Yellow or Green.

Our findings show errors, not only in the recording of discriminators but in points allocation by triage staff when completing TEWS (Step 4 of SATS). As EpiC data collectors correct grossly incorrect TEWS points calculation errors, the TEWS errors determined are likely greater than depicted in this study. In the high‐level general emergency center in Johannesburg, numerical miscalculation accounted for 21.5% of mis‐triage [8]. Poor performance of TEWS points allocation and calculation is inevitable in a paper‐based triage system. Many LMICs are reliant on paper‐based systems that cannot self‐correct, should the accuracy of application be suboptimal. The introduction of an electronic‐based triage system would likely improve this.

There was no significant association between the TBH SATS category and the need for resuscitation, urgent surgical intervention, and 7‐day mortality. Notably, when SATS was correctly applied, no significant association was evident between the above (*p* > 0.05). There are two potential explanations, firstly, the majority of patients had resuscitation initiated at the referring facilities; and secondly, survival bias—those who live the 11‐h average to reach TBH are inherently less complex and less critically injured [19].

SATS was designed for use as an initial triage at the first point of healthcare contact, in LMICs emergency settings for all‐comer undifferentiated conditions. However, TBH Trauma Center is a high‐level specialized trauma center that mostly receives trauma patients transported from other facilities and very few directly from the scene of injury. SATS was designed as a one‐size‐fits‐all triage scale, to have high sensitivity for all conditions but not to be highly specific for any particular conditions, such as patients with PNIs or, more broadly, trauma‐only patients [6]. Secondly, SATS was not designed for triage of patients after they have received initial resuscitation and transfer from another facility [6]. This study was therefore testing SATS in an environment it was not originally designed to be used in, but in which it has been in use for many years.

The cause of the lack of association between SATS category and the need for resuscitation, urgent surgical intervention and 7‐day mortality is likely due to SATS being inappropriate for a specialized trauma center, rather than any problem with SATS, which has been validated in numerous settings [5, 12]. A South African multicenter cohort study that aimed to evaluate trauma scoring tools in predicting in‐hospital mortality has recommended using the Kampala Trauma Score (KTS) due to its performance in predicting 7‐day mortality, with other advantages including straight forward calculations without the need for advanced diagnostic tools for injury classifications. Given the performance of the KTS and its relative ease of use, the TBH Trauma Center is considering implementing this tool to optimize triaging within the center [20].

## 5 Conclusion

Among patients with PNIs, the vast majority of whom were referred to TBH tertiary trauma unit from other facilities, over one quarter (26%) were under‐triaged, largely attributed to the underuse of the clinical discriminators, “Mechanism of injury (high energy transfer)” and “Stabbed neck.” Further, we found that when correctly calculated, SATS final triage colors were not associated with the need for life‐saving interventions and resuscitation among patients with PNIs who were referred to the TBH Trauma Center—this invalidates SATS for the triage of patients with PNIs at a referral center such as the TBH Trauma Center. Our findings suggest that SATS is not optimally applied at TBH for the triage of patients with PNIs in its current application.

## 6 Study Limitations and Assumptions

This was a retrospective study and therefore has inherent risks of error. We assumed all patient folders contained accurate and consistent information. As we used data captured by the EpiC study, we were reliant on accurate data capture from their staff. Patients with missing data were included, with only the incomplete data points being excluded. The small sample size limited a subgroup analysis of PNI patients who were transported directly from the scene to TBH. Recommendations for future research include replication of this study as a prospective study in a larger sample of patients with PNIs, applying the Kampala Trauma Score (KTS) to the same patients with PNIs to determine its effectiveness in predicting in‐hospital mortality within the group, as well as investigation of trauma‐specific triage systems in an electronic format for the TBH Trauma Center.

## Author Contributions

**Thomas Steve Jessop:** conceptualization, formal analysis, investigation, methodology, project administration, visualization, writing – original draft, writing – review and editing. **Lindi Martin:** conceptualization, formal analysis, investigation, methodology, project administration, supervision, visualization, writing – review and editing. **Hendrick J. Lategan:** conceptualization, data curation, methodology, visualization, writing – review and editing. **Julia M. Dixon:** data curation, supervision, writing – review and editing. **Nee‐Kofi Moul‐Millman:** data curation, supervision, writing – review and editing. **Elmin Steyn:** conceptualization, investigation, methodology, project administration, supervision, visualization, writing – original draft, writing – review and editing.

## Ethics Statement

Ethics approval was obtained from the Undergraduate Research Ethics Committee (UREC) of Stellenbosch University (ref no: U23/05/272) and permission to conduct the study was gained from the Western Cape Department of Health. Approval to utilize the EpiC study data was granted by the principal investigators, Dr Lategan and Prof Mould‐Millman.

## Conflicts of Interest

The authors declare no conflicts of interest.
